# Natalizumab concentrations during pregnancy in three patients with multiple sclerosis

**DOI:** 10.1177/13524585211052168

**Published:** 2021-12-21

**Authors:** Alyssa A Toorop, Theo Rispens, Eva MM Strijbis, Bob W van Oosten, Brigit A de Jong, Bernard MJ Uitdehaag, Joep Killestein, Zoé LE van Kempen

**Affiliations:** Department of Neurology, Amsterdam University Medical Centers, Vrije Universiteit Amsterdam, Amsterdam Neuroscience, MS Center Amsterdam, Amsterdam, The Netherlands; Department of Immunopathology, Sanquin Research, Amsterdam, The Netherlands; Landsteiner Laboratory, Academic Medical Centre, University of Amsterdam, Amsterdam, The Netherlands; Biologics Laboratory, Sanquin Diagnostic Services, Amsterdam, The Netherlands; Department of Neurology, Amsterdam University Medical Centers, Vrije Universiteit Amsterdam, Amsterdam Neuroscience, MS Center Amsterdam, Amsterdam, The Netherlands; Department of Neurology, Amsterdam University Medical Centers, Vrije Universiteit Amsterdam, Amsterdam Neuroscience, MS Center Amsterdam, Amsterdam, The Netherlands; Department of Neurology, Amsterdam University Medical Centers, Vrije Universiteit Amsterdam, Amsterdam Neuroscience, MS Center Amsterdam, Amsterdam, The Netherlands; Department of Neurology, Amsterdam University Medical Centers, Vrije Universiteit Amsterdam, Amsterdam Neuroscience, MS Center Amsterdam, Amsterdam, The Netherlands; Department of Neurology, Amsterdam University Medical Centers, Vrije Universiteit Amsterdam, Amsterdam Neuroscience, MS Center Amsterdam, Amsterdam, The Netherlands; Department of Neurology, Amsterdam University Medical Centers, Vrije Universiteit Amsterdam, Amsterdam Neuroscience, MS Center Amsterdam, Amsterdam, The Netherlands

**Keywords:** Multiple sclerosis, natalizumab, personalized dosing, extended interval dosing, pregnancy, concentrations

## Abstract

In women with very active multiple sclerosis (MS), natalizumab can be continued during pregnancy to prevent rebound disease activity. Our aim was to evaluate changes in serum natalizumab trough concentrations during pregnancy. Blood samples of 3 patients were collected before, during, and after pregnancy. Natalizumab trough concentrations gradually decreased during pregnancy. The patient with the lowest trough concentrations during the third trimester was treated with extended interval dosing (EID). After delivery, natalizumab concentrations increased to similar levels as before pregnancy. All patients remained clinically and radiologically stable. MS neurologists should be aware of decreasing natalizumab concentrations during pregnancy, especially in patients with low initial trough concentrations and patients with EID.

## Introduction

Natalizumab, a monoclonal antibody against α_4_ integrin, is an effective therapy for patients with relapsing remitting multiple sclerosis (MS).^1^ Natalizumab is approved for intravenous administration every 4 weeks. However, previous research suggested that extended interval dosing (EID) of natalizumab could be equally effective to 4-week standard interval dosing (SID).^2^ EID of natalizumab could be established by therapeutic drug monitoring, in which infusion intervals are based on serum natalizumab trough concentrations.^2^ Although inter-individual serum natalizumab trough concentrations can vary widely (range: 0.1–112 µg/mL), intra-individual trough concentrations are usually stable within a regular dosing interval.^2,3^

Young women with highly active MS are often treated with natalizumab, including those with a desire for a future pregnancy. Current guidelines recommend continuation of natalizumab during pregnancy in patients with very active MS to prevent rebound disease activity after withdrawal of the drug.^4,5^ Serum concentrations of monoclonal antibodies may however fluctuate during pregnancy, as was demonstrated by Flanagan et al.^6^ Concentrations of vedolizumab, a monoclonal antibody targeting the α_4_β_7_-integrin, decreased during pregnancy (−0.18 µg/L/week, 95% confidence interval (CI) −0.33 to −0.02, *p* = 0.03).^6^ This decrease was small, and dose adjustments or therapeutic drug monitoring was not considered necessary. In previous studies on natalizumab, higher body weight was associated with lower natalizumab concentrations.^2,3^ When natalizumab concentrations are below 1–2 µg/mL, α_4_β_1_-integrin receptor desaturation can occur causing a decrease of natalizumab efficacy.^3,7^ This is especially relevant for patients with EID, who usually have lower trough concentrations of natalizumab compared with patients with SID (mean concentration: 10 vs 30 µg/mL).^2,3^ EID of natalizumab during pregnancy could, on the contrary, be beneficial by minimizing fetal exposure to natalizumab, as mild to moderate hematologic abnormalities such as anemia and thrombocytopenia were observed in infants born after continuation of natalizumab during the third trimester.^8^

Our aim was to evaluate changes in natalizumab trough concentrations during pregnancy in patients with relapsing remitting MS to assess the possible influence of pregnancy on natalizumab pharmacokinetics.

## Methods

In an ongoing pharmacovigilance cohort of patients with MS treated with natalizumab in the MS Center Amsterdam (Amsterdam UMC, location VUMC), blood samples are collected every 3 months and stored in the MS biobank. Of this cohort, patients were selected when natalizumab was continued during the entire pregnancy. Stored blood samples were obtained and serum natalizumab trough concentrations were analyzed at Sanquin Laboratory Amsterdam using a cross-linking assay.^9^ Patient characteristics and information regarding MS were collected. Approval of the institutional ethics committee was obtained (VUMC Ethics committee numbers: 2020.269 and 2016.554). Patients provided written informed consent for the use of data and blood samples from the MS biobank of the MS center Amsterdam.

## Results

Three cases were included. Natalizumab was continued during pregnancy in these patients due to a history of very active MS. Two patients had SID of natalizumab (4-week interval). One of the patients had EID of natalizumab (6-week interval) before and during her pregnancy as part of a current trial (ClinicalTrials.gov Identifier: NCT04225312). Additional measurements of natalizumab trough concentrations were performed in this patient during pregnancy.

**Figure 1.**
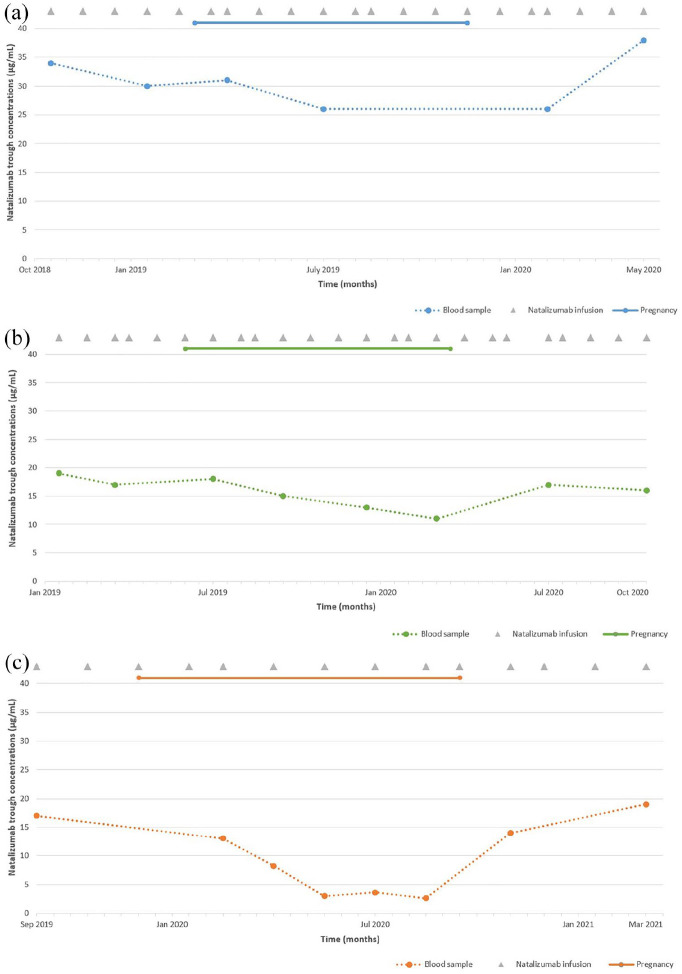
Serum natalizumab trough concentrations of 3 patients with multiple sclerosis during pregnancy. The X-axis represents time in months (0.5 months per step). The Y-axis represents natalizumab trough concentrations in µg/mL. The dots represent the collected blood samples. The duration of pregnancy is depicted by the line above each graph. Natalizumab infusions are depicted as gray triangles. Data on body weight, height, and body mass index were only available after pregnancy. (a) (case 1). Pregnancy between March 2019 and November 2019; duration of pregnancy 37.4 weeks. Start pregnancy: age 32; disease duration 8.6 years; duration of natalizumab treatment 7.2 years; negative for John Cunningham virus (JCV); natalizumab treatment schedule every 4 weeks. Body weight 81 kg, height 171 cm, body mass index 27.7 (August 2020). During the third trimester, no blood samples were collected. (b) (case 2). Pregnancy between June 2019 and March 2020; duration of pregnancy ~40 weeks. Start pregnancy: age 31; disease duration 11.2 years; duration of natalizumab treatment 3.9 years; negative for JCV; natalizumab treatment schedule every 4 weeks. Body weight 73 kg, height 172 cm, body mass index 24.7 (July 2020). The first postpartum measurement (17 µg/mL) was on a one-time 5-week schedule due to patient preference. (c) (case 3). Pregnancy between December 2019 and September 2020; duration of pregnancy 40.7 weeks. Start pregnancy: age 31; disease duration 1.8 years; duration of natalizumab treatment 1.7 years; duration EID of natalizumab 0.4 years; negative for JCV; natalizumab treatment schedule every 6 weeks. Body weight 82 kg, height 184 cm, body mass index 24.2 (November 2020) The second measurement during the second trimester (3.0 µg/mL) was on a one-time 7-week schedule due to patient preference. The first postpartum measurement (14 µg/mL) was on a one-time 5-week schedule due to low natalizumab concentrations during the third trimester.

In all patients, natalizumab trough concentrations gradually declined during pregnancy (Figure 1). Natalizumab trough concentrations in these 3 patients ranged from 17–34 µg/mL before pregnancy, 13–31 µg/mL during the first trimester, 3–26 µg/mL during the second trimester, and 2.6–13 µg/mL (*n* = 2) during the third trimester. The patient with EID of natalizumab had the lowest trough concentration of 2.6 µg/mL during the third trimester. After delivery, natalizumab trough concentrations increased to similar concentrations as before pregnancy (range: 19–38 µg/mL). All patients remained clinically stable (no new neurological symptoms evaluated by a neurologist, lasting more than 24 hours and caused by MS) and radiologically stable (no new or enlarged T2 lesions on yearly brain magnetic resonance imaging (MRI) scans) during pregnancy and up to 1 year after delivery.

## Discussion

These cases illustrate decreasing serum natalizumab trough concentrations during pregnancy. In the patient with EID, trough concentrations during the third trimester were roughly 85% lower compared with trough concentrations before and after pregnancy. The lowest concentration of 2.6 µg/mL was close to the assumed therapeutic cut-off of 1–2 µg/mL.^7^ In both patients with SID, initial trough concentrations before pregnancy were higher and natalizumab concentrations remained adequate during pregnancy.

During pregnancy, volume of distribution increases due to increased total body water and increased body fat.^10^ Since monoclonal antibodies have a relatively limited volume of distribution within plasma and extracellular fluid, major changes in concentrations are not expected.^6^ However, in a study investigating the pharmacokinetics of monoclonal antibodies during pregnancy, vedolizumab concentrations were lower during pregnancy, similar to our observations, possibly by pregnancy-related alterations in drug clearance.^6^ In a case series of patients using natalizumab during the third trimester, natalizumab concentrations of 5 mother–infant pairs after delivery were higher in mothers and infants with more frequent and more recent exposure to natalizumab.^8^ In one mother, natalizumab concentrations were low (1.6 µg/mL) without the presence of natalizumab antibodies. Although previous natalizumab concentrations were not reported, the low natalizumab concentration in this patient could be due to inter-individual differences in natalizumab concentrations and possibly lower natalizumab concentrations during her pregnancy.

Pregnancies can effectuate a protective function on MS disease activity. Nonetheless, the risk of relapses even during pregnancy can be higher in patients discontinuing natalizumab due to rebound disease activity.^4^ This can be a challenging decision because data on infant outcomes born after natalizumab exposure are limited.^5,8^ Reducing natalizumab exposure with EID of natalizumab in women who continue natalizumab during pregnancy could possibly reduce harmful effects on the fetus. Therapeutic drug monitoring of natalizumab trough concentrations is currently not being used for patients on SID. However, since patients on EID of natalizumab usually have lower trough concentrations compared with patients on SID, therapeutic drug monitoring could be relevant for this patient group.

In conclusion, natalizumab concentrations can decrease during pregnancy. MS neurologists should be aware of decreasing natalizumab concentrations during pregnancy, especially in patients with low initial trough concentrations and patients with EID.
